# Time restricted eating and exercise training before and during pregnancy for people with increased risk of gestational diabetes: single centre randomised controlled trial (BEFORE THE BEGINNING)

**DOI:** 10.1136/bmj-2024-083398

**Published:** 2025-09-09

**Authors:** MA Jafar Sujan, Hanna MS Skarstad, Guro Rosvold, Stine L Fougner, Turid Follestad, Kjell Å Salvesen, Trine Moholdt

**Affiliations:** 1 Department of Circulation and Medical Imaging, Norwegian University of Science and Technology, Trondheim, Norway; 2 Department of Women’s Health, St Olav’s Hospital, Trondheim University Hospital, Trondheim, Norway; 3 Department of Clinical and Molecular Medicine, Norwegian University of Science and Technology, Trondheim, Norway; 4 Department of Endocrinology, St Olav’s Hospital, Trondheim University Hospital, Trondheim, Norway; 5 Department of Public Health and Nursing, Norwegian University of Science and Technology, Trondheim, Norway

## Abstract

**Objective:**

To determine the effect of a prepregnancy lifestyle intervention on glucose tolerance in people at higher risk of gestational diabetes mellitus.

**Design:**

Single centre randomised controlled trial (BEFORE THE BEGINNING).

**Setting:**

University hospital in Trondheim, Norway.

**Participants:**

167 participants with at least one risk factor for gestational diabetes mellitus who contemplated pregnancy.

**Intervention:**

The participants were randomly allocated (1:1) to a lifestyle intervention or a standard care control group. The intervention consisted of exercise training and time restricted eating, started before pregnancy and continued throughout pregnancy. Exercise volume was set using a physical activity metric that translates heart rate into a score (personal activity intelligence, PAI), with the goal of ≥100 weekly PAI points. Time restricted eating involved consuming all energy within ≤10 hours/day for at least five days a week.

**Main outcome measures:**

Two hour plasma glucose level in an oral glucose tolerance test at gestational week 28. The primary analysis used an intention-to-treat principle.

**Results:**

167 participants were enrolled from 2 October 2020 to 12 May 2023: 84 in the intervention group and 83 in the control group, out of whom 111 became pregnant (56 in intervention group and 55 in control group). One participant in the intervention group was excluded from the analysis because of prepregnancy diabetes. Pregnancy data from one participant in the control group were excluded from the analysis because of twin pregnancy. The intervention had no significant effect on two hour plasma glucose level in an oral glucose tolerance test at gestational week 28 (mean difference 0.48 mmol/L, 95% confidence interval −0.05 to 1.01, P=0.08). In the prepregnancy period, 31/83 participants (37%) in the intervention group adhered to prespecified criteria, whereas 24/55 participants (44%) in the intervention group who became pregnant fulfilled these criteria. During the prepregnancy period, the average eating window was 9.9 hours/day (standard deviation 1.2) and the average number of weekly PAI points was 111 (standard deviation 54), but the adherence to both intervention components decreased during pregnancy.

**Conclusions:**

A combination of time restricted eating and exercise training started before and continued throughout pregnancy had no significant effect on glycaemic control in late pregnancy.

**Trial registration:**

ClinicalTrials.gov NCT04585581.

## Introduction

Gestational diabetes mellitus (GDM) is defined as hyperglycaemia first diagnosed during pregnancy and affects approximately one out of seven live births globally.^1^ Risk factors for GDM include having a high body mass index and excessive gestational weight gain, older age, GDM in a previous pregnancy, a family history of diabetes, and non-European ethnicity.^1^ Chronic insulin resistance as a result of a complex interplay of these genetic, environmental, and behavioural risk factors aggravates physiological insulin resistance in the second half of pregnancy, leading to pancreatic β cell dysfunction, raised glucose levels, and eventually GDM.^2^ Although hyperglycaemia usually resolves after delivery, GDM is associated with an increased risk of several adverse consequences later in life, including recurrence of GDM in later pregnancies, type 2 diabetes, metabolic syndrome, and cardiovascular diseases.^3^ ^4^ ^5^ ^6^ Children of mothers with GDM are at increased risk of cardiac dysfunction at birth, childhood obesity, and future diabetes mellitus, therefore continuing the intergenerational cycle of obesity and diabetes.^7^ ^8^ ^9^ ^10^ ^11^

Conventional lifestyle recommendations in pregnancy include moderate intensity exercise for at least 150 minutes per week and a healthy diet.^12^ However, many pregnant people fail to meet these recommendations, and it might be difficult to change lifestyle because of biological changes in pregnancy and owing to concerns about the effects on the growing fetus.^13^ ^14^ ^15^ The Finnish Gestational Diabetes Prevention Study (RADIEL) showed that a diet and exercise intervention started before 20 weeks of gestation could reduce the incidence of GDM by 39% in those at high risk.^16^ In contrast, some large lifestyle intervention trials, such as the UPBEAT study^17^ and the LIMIT trial,^18^ did not show the effectiveness of a diet and exercise intervention in pregnancy for GDM prevention. Most clinical trials of lifestyle interventions in pregnancy have started the intervention around 16-20 weeks of gestation, leaving a missed window of opportunity to implement lifestyle changes and improve glycaemic control.^19^ Because prepregnancy patterns of physical activity are associated with exercise during pregnancy^20^ and prepregnancy healthy dietary habits are associated with a lower risk of GDM,^21^ the prepregnancy period can be a window of opportunity to make favourable changes that can improve maternal and fetal outcomes. Over the past few years, several systematic reviews and meta-analyses have concluded that prepregnancy lifestyle interventions are necessary to improve adherence and reduce the risk of GDM and related short and longer term adverse outcomes.^22^ ^23^ ^24^ However, evidence on the specific components of prepregnancy interventions and their effectiveness remains scarce. The Prepare randomised controlled trial indicated that a weight loss intervention before pregnancy could reduce the rates of GDM diagnosis in early pregnancy,^25^ but not at gestational weeks 24-28.^26^

Alternative diet and exercise intervention strategies, such as time restricted eating and high intensity interval training (HIIT), improve glycaemic control and cardiometabolic outcomes in non-pregnant people with cardiometabolic disorders.^27^ ^28^ ^29^ ^30^ ^31^ As such, seven weeks of combined time restricted eating and HIIT decreased glycated haemoglobin (HbA1c), total body mass, fat mass, and visceral fat mass in reproductive aged women with overweight or obesity.^28^ Data on the effects of time restricted eating in pregnancy are scarce. We recently showed that it was feasible to consume all energy within a maximum of 10 hours/day for five weeks in the second and third trimesters of pregnancy, even though no effect on the measured cardiometabolic outcomes was found.^32^ However, observational data indicate that longer night fasting duration is associated with improved fasting glucose in those who are pregnant.^33^ Recent publications show that HIIT is safe and enjoyable, and relatively easy to adhere to during pregnancy.^34^ ^35^ ^36^ In the BEFORE THE BEGINNING trial, we hypothesised that combined time restricted eating and exercise training started before pregnancy and continued throughout pregnancy would improve maternal glucose tolerance by gestational week 28 in people at increased risk of GDM.

## Methods

### Study design

**Fig 1:**
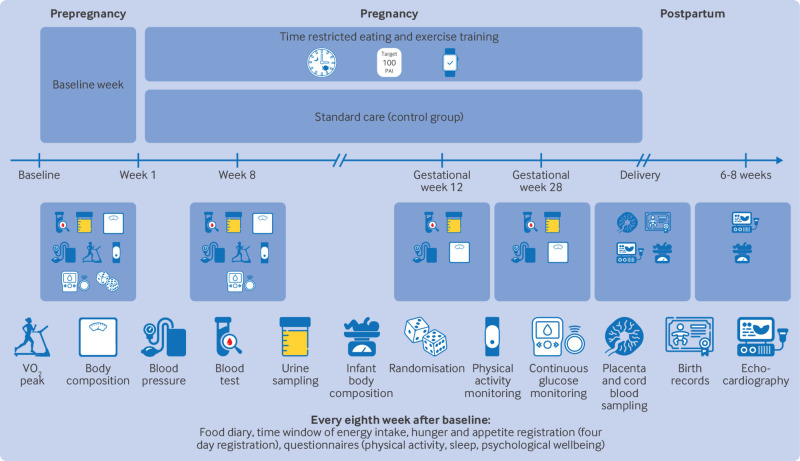
Study design. After baseline assessments, participants were randomly allocated (1:1) to lifestyle intervention or standard care control group. Intervention consisted of exercise training and time restricted eating, started before pregnancy and continued throughout pregnancy. Amount of exercise was set using heart rate based physical activity metric (personal activity intelligence, PAI), with goal of ≥100 weekly PAI points. Time restricted eating involved consuming all energy within ≤10 hours/day for at least five days a week. Further assessments were performed eight weeks after baseline in prepregnancy period, and at gestational weeks 12 and 28. Pregnancy and birth outcomes were collected from hospital records after delivery. Neonatal outcomes were assessed within 72 hours after birth and 6-8 weeks after birth

BEFORE THE BEGINNING was a single centre, randomised controlled trial with two parallel groups: an intervention group and a control group (fig 1). The trial was undertaken at the Norwegian University of Science and Technology (NTNU) in Trondheim, Norway, in collaboration with St Olav’s Hospital, Trondheim, Norway. The trial was registered in ClinicalTrials.gov (NCT04585581) on 25 September 2020. A detailed study protocol has been published previously.^37^

### Recruitment and participants

We advertised the trial on social media, hospital and university websites, in local stores, and public places. We also sent electronic invitations to participate in the trial to all women aged 20-35 years in Trondheim and the surrounding area using information obtained from the national population register. We included those aged 18-39 years who were contemplating pregnancy within the next six months, who understood oral and written Norwegian or English, and who had at least one of the following risk factors: body mass index ≥25, GDM in a previous pregnancy, close relative with diabetes (parents, siblings, or children with diabetes), fasting plasma glucose >5.3 mmol/L, previous newborn >4.5 kg, or non-European ethnicity (one or both parents originating from an area outside Europe). Exclusion criteria were ongoing pregnancy, trying to conceive for at least six cycles at study entry, known diabetes (type 1 or 2), shift work that included night shifts for more than two days a week, previous hyperemesis, known cardiovascular diseases, high intensity exercise more than twice a week in the past three months, habitual eating window ≤12 hours/day, bariatric surgery, or any other reason that according to the researchers makes the potential participant ineligible.

### Randomisation and blinding

Baseline assessments were performed before we randomly allocated the participants (1:1) to the intervention group or a standard care control group, stratified by GDM in a previous pregnancy (yes or no). The study personnel used WebCRF3, a computer random number generator developed and administered at the Clinical Research Unit (Klinforsk, NTNU/St Olav’s Hospital, Trondheim, Norway) to randomly allocate participants using various block sizes. The participants were enrolled and assigned to interventions by the principal investigator (TM), a study coordinator (GR), or PhD students (MAJS and HMSS). The randomisation sequence was generated by a technician at Klinforsk and concealed until interventions were assigned. Neither participants nor study personnel were masked.

### Intervention and adherence

The intervention consisted of time restricted eating and exercise training, and spanned from inclusion before pregnancy and throughout pregnancy. We counselled the participants in the intervention group to restrict their daily time window of energy intake to ≤10 hours, ending no later than 7 pm, for a minimum of five days per week throughout the study period. On the “off” days, the participants could choose their time window for energy intake. We gave no advice about dietary composition or the amount of energy the participants should consume, and they could consume non-energy drinks outside the time window.

The exercise programme was based on personal activity intelligence (PAI), which is a physical activity metric that translates heart rate during physical activity into a score.^38^ We instructed the participants to obtain ≥100 weekly PAI points throughout the study period because this amount of physical activity is associated with higher cardiorespiratory fitness and a decreased cardiovascular disease risk.^38^ ^39^ High intensity exercise and therefore higher heart rates give substantially more PAI points than low to moderate intensity exercise. The participants in the intervention group wore smartwatches (Amazfit GTS or Polar Ignite 2) throughout the study period. These watches shared PAI data with the research team.

Exercise training consisted of endurance exercise with the aim of high intensity and was mainly unsupervised. We provided participants in the intervention group with a brochure suggesting various HIIT sessions to complete at home (supplementary file 1). Participants were free to choose whether to exercise indoors or outdoors, and if they wanted to use any cardio machines (eg, treadmill, stationary bike). Once pregnant, we advised participants to choose between repeated short (30 seconds) work bouts at high intensity with low to moderate intensity periods in between, or longer work periods with an intensity up to 85% of heart rate maximum. Participants were invited for supervised exercise sessions twice after baseline—after two and four weeks. Additionally, participants could ask for extra support and more supervised sessions at any time during the intervention period. The supervised sessions were undertaken on a treadmill or a stationary bike in our training facility. Participants in the control group received standard care and were asked to continue with their habitual physical activity and dietary intake.

We asked all participants to report their daily time window of energy intake for four days (three weekdays and one weekend day) every eighth week in a printed study handbook. We categorised participants in the intervention group as adherent to time restricted eating if they reported a time window for energy intake of ≤10 hours on at least two of these four days and adherent to exercise training if they earned and maintained ≥75 PAI points per rolling week. We sent reminders to all participants by text message to complete dietary reporting and contacted the participants in the intervention group to offer additional supervised exercise sessions if they were not reaching the PAI target.

### Experimental procedures and outcome measures

Assessments of the participants were undertaken twice during the prepregnancy period and twice during pregnancy. The first week after allocation to groups consisted of baseline recordings, followed by visits in the laboratory after eight weeks, and in gestational weeks 12 and 28. Participants who did not become pregnant within six months of inclusion were excluded from the study, but their prepregnancy data were included in the intention-to-treat analysis (changed from 12 months; see modifications to the protocol after start of trial). When spontaneous abortion occurred, we adjusted the period to allow participants to continue in the study by a four week extension added to the number of weeks the participants were pregnant before abortion.

#### Primary outcome measure

The primary outcome measure was two hour plasma glucose concentration in a 75 g oral glucose tolerance test at gestational week 28. After an overnight fast (≥10 hours) and no exercise for ≥24 hours, participants consumed a premade drink of 75 g glucose diluted in 250 mL water (Glucosepro, Finnamedical, Finland) within five minutes. Using an indwelling catheter, we collected venous blood before the oral glucose tolerance test, with subsequent collections at 30, 60, 90, and 120 minutes after ingestion of glucose.

#### Secondary outcome measures

The trial has several secondary outcomes, as specified in the study protocol.^37^ Here we report the main secondary maternal cardiometabolic outcomes and will report the remaining secondary outcomes in separate publications. Secondary outcome measures were assessed twice during prepregnancy (at baseline and after eight weeks) and twice during pregnancy (at gestational weeks 12 and 28), if not otherwise specified. Fasting plasma glucose, blood lipids, and HbA1c were analysed immediately after blood sampling at St Olav’s Hospital laboratory, following local standardised procedures. GDM was recorded at gestational weeks 12 and 28, and diagnosed according to the World Health Organization (WHO) 2013 criteria: fasting plasma glucose 5.1-6.9 mmol/L or two hour plasma glucose 8.5-11.0 mmol/L, or both, after a 75 g glucose load.^40^ We measured fasting, 30 minute, and 120 minute insulin concentrations in thawed serum samples with enzyme linked immunosorbent assay (ELISA, IBL-International, Hamburg, Germany) according to the manufacturer’s protocol using a DS2 ELISA processing system (Dynex technologies, Virginia, USA) at the research laboratories at the Department of Circulation and Medical Imaging, NTNU. The area under the curve (AUC) and incremental AUC (iAUC) from glucose concentrations were calculated from venous blood sampling obtained every 30 minutes during the oral glucose tolerance test.^41^ We calculated homoeostasis model assessment of insulin resistance (HOMA2-IR) and pancreatic β cell function (HOMA2-β) using the online HOMA2 calculator: https://www.dtu.ox.ac.uk/homacalculator/index.php.^42^ We also estimated the insulin sensitivity index (ISI_0,120_),^43^ insulinogenic index during the first 30 minutes of the oral glucose tolerance test,^44^ and β cell function (AUC_ins_/AUC_glu_).^45^

Weight and body composition were estimated in the morning after overnight fasting using bioelectrical impedance analysis (Inbody 720, Biospace CO, Korea), with participants wearing light clothes and standing barefoot. Additionally, waist circumference was measured using a measuring tape at the level of the navel, in a standing position. We used an automatic blood pressure device (Welch Allyn, Germany) to measure blood pressure and resting heart rate on the participants’ left arm after they had rested in a seated position for 15 minutes. The average of three measurements taken one minute apart is reported. We asked the participants to register their diet in an online food diary (Fatsecret app) for four days (three weekdays and one weekend day) every eighth week. They also completed the International Physical Activity Questionnaire^46^ every eighth week throughout the study period.

#### Modifications to protocol after start of trial

From June 2021, we offered supervised exercise sessions for the participants in the intervention group. From November 2022, we started identifying eligible participants using population data from the Norwegian Tax Administration and sending out electronic participant invitations to reach a broader target population. Concurrently, we added bariatric surgery and any other reason that according to the researchers makes the potential participant ineligible to undergo one or both interventions (eg, traumatic foot injury, anorexia or bulimia) as exclusion criteria. From December 2022, we removed planned assisted fertilisation with female factor reason from the exclusion criteria. The changes in the inclusion criteria were made to account for challenges that arose during the screening process. Also in December 2022, we changed the maximum time before pregnancy from 12 months to six months to allow for the trial to be terminated in time for us to analyse the data within the project period. In March 2023, the required number of total participants was reduced from 260 to 200 based on the revised sample size calculation described below. In June 2023, we changed from Amazfit GTS (Huami, China) to Polar Ignite 2 (Polar, Finland) smartwatch and from Zepp and Memento to Polar Flow and Mia app to improve recording of PAI points.

#### Sample size

The primary outcome measure was two hour plasma glucose level in a 75 g oral glucose tolerance test at gestational week 28. We considered a difference between the intervention group and the control group of 1.0 mmol/L as the minimally clinically relevant difference based on findings from the HAPO study.^7^ We also used the observed standard deviation in two hour plasma glucose concentrations during the oral glucose tolerance test from HAPO in the calculations. Sample size calculation for a two sided t test to detect a 1.0 mmol/L difference between the groups, using a standard deviation of 1.3, a power of 0.90, and a significance level of 0.05, yields 37 participants in each group at gestational week 28. To allow for an expected exclusion from the study because of not conceiving within the study period (about 50%)^47^ yielding 74 per group, further dropout during the study period (10-20%), yielding 93 per group, and to increase statistical power for secondary analyses, we initially wanted to include 260 participants in the trial. However, we concluded participant recruitment after reaching 167 participants because at that point, we had 47 participants per group at gestational week 12, which allowed for an anticipated 20% dropout during pregnancy.^37^

#### Statistical analysis

We used linear mixed models to estimate differences in primary and secondary continuous outcomes between groups, with time and the interaction between time and group as fixed effects variables, and subject (participant ID) as random effect.^48^ Because no systematic baseline differences between the groups are expected in randomised controlled trials, the main effect of group was not included, so that the means at baseline were constrained to be equal in these models. We report estimated effects in the intervention group compared with the control group, with corresponding 95% confidence intervals and P values. For outcomes obtained only during the pregnancy period (variables using data from the oral glucose tolerance test at gestational weeks 12 and 28), we included the main effect of group in the linear mixed model.

We checked the normality of residuals by visually inspecting QQ plots. For variables that were not normally distributed, bias corrected and accelerated bootstrap confidence intervals based on 3000 bootstrap samples were calculated. Additionally, we used Fisher’s exact test to compare GDM prevalence at gestational week 12 and χ^2^ test at gestational week 28, and Student’s t test to compare time to pregnancy between groups. For our primary outcome measure, we considered a P value <0.05 to indicate statistically significant results. For the secondary outcome measures, we used a significance level of 0.01 to give some protection against false positives owing to several comparisons. We also performed prespecified per protocol analyses, in which we included only participants in the intervention group who obtained ≥75 weekly PAI points and reported a ≤10 hour time window for energy intake on at least two of four days in the handbook before pregnancy. Statistical analyses were performed using IBM SPSS Statistics 29.0 and STATA MP version 18.

### Patient and public involvement

In the planning phase, we invited users to discuss the study one month before applying to the regional ethical committee for approval. We arranged a one hour interactive digital workshop with participant representatives (reproductive age women with overweight or obesity) to discuss relevant topics or issues related to participation and long term adherence. Among the topics we discussed were potential barriers to participation in diet-exercise interventions, how to engage participants and keep them engaged, how to increase motivation, use of digital methods to collect exercise and diet data, and recruitment strategies. Four months after including the first participant, we organised another interactive workshop in which we discussed how to increase participant recruitment and adherence. Based on input from the participant representatives, we established a Facebook group for planning exercise sessions together with other participants. Additionally, we offered supervised exercise training for interested participants at our training facilities through the Facebook group. Participant representatives were not involved in the recruitment process. In February 2023, we had another interactive meeting with participants who completed the study to discuss their views on exercise training during pregnancy and follow-up before and during pregnancy.

## Results

### Participants

**Fig 2:**
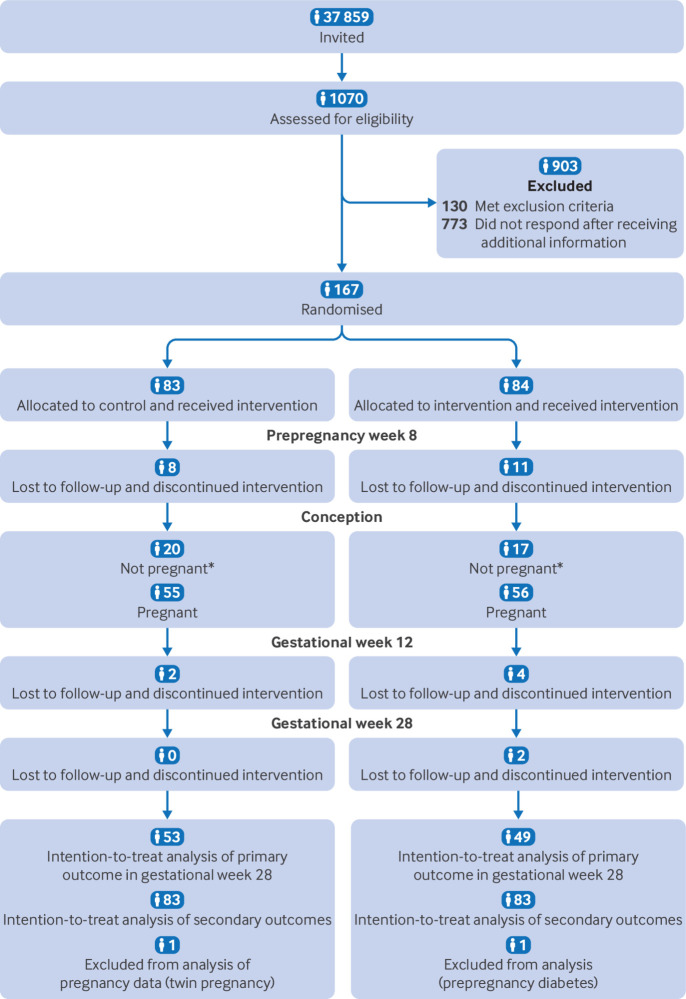
Flowchart of participants. *Participants who did not conceive within specified time were excluded from further participation in trial, but their prepregnancy data were included

We randomised 167 participants (84 in the intervention group and 83 in the control group) between 2 October 2020 and 12 May 2023 (fig 2). We ended inclusion of new participants when 47 participants in each group reached gestational week 12, according to our sample size calculations. Within the specified time, 111 participants became pregnant (56 in intervention group and 55 in control group). We excluded data from one participant in the intervention group because of prepregnancy diabetes, so that data from 166 participants were included in the intention-to-treat analyses and 110 were included in the analysis of time to pregnancy. Pregnancy data from one participant in the control group were excluded from the analysis because of twin pregnancy, leaving data from 109 participants in the analyses of pregnancy specific data (fig 2). Time to pregnancy did not differ significantly between groups, with a mean of 112 days (standard deviation 105) in the intervention group and 84 days (69) in the control group (P=0.10). Table 1 shows the baseline characteristics of the participants.

**Table 1:** Baseline characteristics of participants according to group allocation

Characteristics	Control (n=83)	Intervention (n=83)
Age, years	30.3 (3.2)	30.2 (3.1)
Weight, kg	81.5 (13.2)	81.1 (16)
Body mass index	29.1 (4.5)	29.2 (4.9)
Waist circumference, cm	94.5 (11.8)	93.0 (12.3)
Muscle mass, kg	27.7 (3.6)	27.9 (4.3)
Fat mass, kg	31.7 (9.9)	30.8 (11)
Fat percentage, %	37.9 (7.2)	37.0 (7.6)
Visceral fat area, cm^2^	153 (52)	146 (56)
Systolic blood pressure, mm Hg	121 (10)	118 (8)
Diastolic blood pressure, mm Hg	79 (7)	79 (6)
Resting heart rate, bpm	72 (11)	71 (11)
HbA1c, mmol/mol	34.3 (3.1)	34.1 (2.8)
Fasting glucose, mmol/L	5.0 (0.4)	4.9 (0.4)
Total cholesterol, mmol/L	4.5 (0.7)	4.6 (0.8)
HDL cholesterol, mmol/L	1.4 (0.3)	1.4 (0.3)
LDL cholesterol, mmol/L	3.0 (0.9)	3.1 (0.8)
Triglycerides, mmol/L	1.0 (0.5)	1.0 (0.5)
Fasting insulin, μIU/mL	19.1 (11.7)	17.1 (10.4)
HOMA2-IR	2.4 (1.4)	2.2 (1.2)
HOMA2-B	168.2 (59.2)	164.9 (69.2)
Parity, total number of births, n (%)		
0	45 (55)	47 (58)
1	30 (37)	30 (37)
2	7 (8)	4 (5)
Education level, n (%)		
Compulsory schooling	0 (0)	1 (1)
Completed upper secondary school	8 (10)	10 (12)
Completed university education, <4 years	28 (34)	23 (28)
Completed university education, ≥4 years	46 (56)	47 (58)
Ethnic origin, n (%)		
Europe	74 (89)	72 (87)
Africa and Middle East	3 (4)	1 (1)
Asia	4 (5)	6 (7)
North America	1 (1)	1 (1)
Latin America	1 (1)	3 (4)
Reason for inclusion,* n (%)		
Body mass index ≥25	74 (89)	68 (82)
GDM in previous pregnancy	1 (1)	2 (2)
Family history of diabetes	20 (24)	24 (29)
Previous newborn >4.5 kg	1 (1)	1 (1)

Data are presented as means of observed values (standard deviations) unless indicated otherwise. Number of participants with missing data in each group is 0-3 for all variables except for waist circumference (5 missing in intervention group and 8 in control group).

GDM=gestational diabetes mellitus; HbA1c=glycated haemoglobin; HDL=high density lipoprotein; HOMA2-B=homoeostatic model assessment of β cell function; HOMA2-IR=homoeostatic model assessment of insulin resistance; LDL=low density lipoprotein.

* Some fulfilled more than one reason.

### Primary outcome and secondary glycaemic outcomes in pregnancy

**Fig 3:**
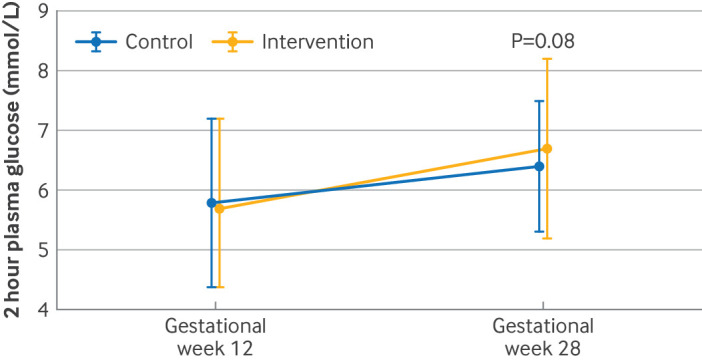
Blood glucose two hours after ingestion of 75 g glucose in oral glucose tolerance test at gestational weeks 12 and 28 according to group. Data are observed means and standard deviations for intention-to-treat population. P value calculated for between group differences using linear mixed model

There was no statistically significant difference between the intervention and control groups in two hour plasma glucose concentrations during a 75 g oral glucose tolerance test (mean difference 0.48 mmol/L, 95% confidence interval −0.05 to 1.01, P=0.08) at gestational week 28 (table 2, fig 3). The intervention did not significantly improve secondary glycaemic outcomes (fasting glucose, fasting insulin, HbA1c, HOMA2-IR, and HOMA2-B) compared with the control group. No statistically significant between group differences were found in glycaemic outcomes obtained only during pregnancy (AUC, iAUC, insulinogenic index during the first 30 minutes of oral glucose tolerance test, and ISI_0,120_; supplementary table 1). At gestational week 12, 3/51 participants (5.9%) in each group fulfilled the criteria for GDM diagnosis (P=1.00). The corresponding numbers at gestational week 28 were 8/49 participants (16.3%) in the intervention group and 6/52 participants (11.5%) in the control group (P=0.57; supplementary table 1).

**Table 2:** Intention-to-treat analyses

Outcome and time	Control, mean (SD)	Intervention, mean (SD)	Difference (intervention−control)	
			Estimated effect (95%CI)	P value
**Two hour plasma glucose during OGTT, mmol/L (n=102)**				
Gestational week 12	5.8 (1.4)	5.7 (1.3)	—	—
Gestational week 28	6.4 (1.1)	6.7 (1.5)	0.48 (−0.05 to 1.01)	0.08
**Fasting blood glucose, mmol/L (n=166)**				
Baseline	5.0 (0.4)	4.9 (0.4)	—	—
Prepregnancy week 8	5.1 (0.4)	5.0 (0.3)	0.0 (−0.2 to 0.1)	0.39
Gestational week 12	4.5 (0.3)	4.5 (0.3)	0.0 (−0.1 to 0.1)	0.64
Gestational week 28	4.5 (0.4)	4.5 (0.5)	0.0 (−0.1 to 0.2)	0.43
**HbA1c, mmol/mol (n=166)**				
Baseline	34.3 (3.1)	34.1 (2.8)	—	—
Prepregnancy week 8	34.5 (3.2)	33.9 (2.5)	−0.4 (−1.1 to 0.2)	0.21
Gestational week 12	32.7 (2.3)	33.3 (2.4)	0.5 (−0.2 to 1.2)	0.14
Gestational week 28	32.3 (2.8)	32.0 (3.19)	−0.2 (−0.9 to 0.5)	0.52
Fasting insulin, μIU/mL* (n=165)				
Baseline	19.1 (11.7)	17.1 (10.4)	—	—
Prepregnancy week 8	19.3 (9.7)	16.3 (8.6)	−2.0 (−4.4 to 0.5)	0.12
Gestational week 12	12.8 (10.7)	12.3 (9.1)	0.1 (−3.8 to 4.0)	0.96
Gestational week 28	19.4 (12.2)	18.6 (11.0)	−0.8 (−5.0 to 3.4)	0.72
HOMA2-B* (n=166)				
Baseline	167.8 (58.9)	164.9 (69.2)	—	—
Prepregnancy week 8	171.2 (59.2)	155.0 (51.6)	−13.8 (−30.1 to 2.5)	0.10
Gestational week 12	153.8 (81.4)	155.8 (68.7)	4.6 (−24.1 to 33.2)	0.76
Gestational week 28	212.4 (84.9)	205.2 (70.0)	−7.7 (−35.1 to 19.6)	0.58
HOMA2-IR* (n=166)				
Baseline	2.4 (1.4)	2.2 (1.2)	—	—
Prepregnancy week 8	2.4 (1.2)	2.1 (1.1)	−0.2 (−0.5 to 0.1)	0.21
Gestational week 12	1.6 (1.2)	1.5 (1.1)	0.1 (−0.4 to 0.5)	0.79
Gestational week 28	2.3 (1.4)	2.3 (1.3)	0.0 (−0.5 to 0.5)	0.88
**Total cholesterol, mmol/L (n=166)**				
Baseline	4.6 (0.7)	4.6 (0.8)	—	—
Prepregnancy week 8	4.5 (0.7)	4.7 (0.8)	0.3 (0.0 to 0.5)	0.03
Gestational week 12	4.4 (0.7)	4.4 (0.8)	0.0 (−0.3 to 0.2)	0.81
Gestational week 28	5.9 (1.2)	5.8 (1.1)	−0.1 (−0.3 to 0.2)	0.57
**LDL cholesterol, mmol/L (n=166)**				
Baseline	3.0 (0.9)	3.1 (0.8)	—	—
Prepregnancy week 8	2.9 (0.8)	3.1 (0.8)	0.2 (0.0 to 0.4)	0.09
Gestational week 12	2.8 (0.7)	2.8 (0.8)	0.0 (−0.3 to 0.2)	0.84
Gestational week 28	3.9 (1.2)	3.9 (1.1)	−0.1 (−0.4 to 0.1)	0.40
**HDL cholesterol, mmol/L (n=166)**				
Baseline	1.4 (0.3)	1.4 (0.3)	—	—
Prepregnancy week 8	1.4 (0.3)	1.5 (0.3)	0.0 (0.0 to 0.1)	0.38
Gestational week 12	1.6 (0.4)	1.6 (0.2)	0.0 (−0.1 to 0.1)	0.49
Gestational week 28	1.8 (0.4)	1.8 (0.3)	0.0 (−0.1 to 0.1)	0.82
**Triglycerides, mmol/L (n=166)**				
Baseline	1.0 (0.5)	0.9 (0.5)	—	—
Prepregnancy week 8	1.0 (0.5)	0.9 (0.4)	0.0 (−0.2 to 0.1)	0.83
Gestational week 12	1.1 (0.4)	1.1 (0.4)	0.0 (−0.1 to 0.2)	0.55
Gestational week 28	2.0 (0.7)	1.9 (0.6)	0.0 (−0.2 to 0.1)	0.74
**Weight, kg (n=166)**				
Baseline	81.5 (13.2)	81.1 (16.0)	—	—
Prepregnancy week 8	81.6 (12.5)	78.9 (14.8)	−0.9 (−2.0 to 0.3)	0.15
Gestational week 12	79.5 (12.9)	78.8 (15.2)	−0.9 (−2.2 to 0.3)	0.15
Gestational week 28	86.8 (12.3)	85.1 (15.4)	−2.0 (−3.3 to −0.8)	0.002
**Fat mass, kg (n=166)**				
Baseline	31.6 (9.8)	30.8 (11.0)	—	—
Prepregnancy week 8	31.2 (9.5)	28.8 (9.7)	−0.8 (−1.8 to 0.2)	0.10
Gestational week 12	30.6 (9.5)	29.5 (10.7)	−0.5 (−1.5 to 0.6)	0.41
Gestational week 28	34.2 (9.0)	32.5 (10.8)	−1.5 (−2.5 to −0.4)	0.008
**Fat percentage, % (n=166)**				
Baseline	37.9 (7.2)	37.0 (7.6)	—	—
Prepregnancy week 8	37.6 (7.0)	35.7 (6.6)	−0.6 (−1.4 to 0.2)	0.14
Gestational week 12	37.7 (7.0)	36.5 (7.4)	−0.2 (−1.0 to 0.7)	0.73
Gestational week 28	38.8 (6.0)	37.4 (7.2)	−0.7 (−1.5 to 0.2)	0.14
**Visceral fat area, cm^2^ (n=166)**				
Baseline	153.6 (52)	146 (57)	—	—
Prepregnancy week 8	151 (50)	135 (50)	−5.1 (−10.9 to 0.7)	0.09
Gestational week 12	148 (51)	136 (54)	−2.3 (−8.6 to 4.1)	0.48
Gestational week 28	165 (47)	153 (54)	−6.2 (−12.5 to 0.1)	0.05
**Muscle mass, kg (n=166)**				
Baseline	27.7 (3.6)	27.9 (4.3)	—	—
Prepregnancy week 8	28.0 (3.6)	27.8 (4.2)	0.0 (−0.3 to 0.3)	0.86
Gestational week 12	27.0 (3.2)	27.2 (4.1)	−0.2 (−0.5 to 0.2)	0.32
Gestational week 28	29.1 (3.1)	29.1 (4.2)	−0.3 (−0.7 to 0.0)	0.06
**Waist circumference, cm (n=162)**				
Baseline	94.5 (11.8)	93.0 (12.3)	—	—
Prepregnancy week 8	93.9 (11.2)	92.0 (11.5)	−0.5 (−2.9 to 1.8)	0.66
Gestational week 12	95.1 (11.5)	94.9 (12.1)	0.6 (−1.8 to 3.1)	0.61
Gestational week 28	107.3 (8.4)	105.9 (10.6)	−1.5 (−4.0 to 1.0)	0.24
**Systolic blood pressure, mm Hg (n=166)**				
Baseline	121 (10)	118 (8)	—	—
Prepregnancy week 8	119 (8)	117 (8)	0.2 (−2.4 to 2.7)	0.90
Gestational week 12	114 (10)	110 (8)	−2.2 (−5.0 to 0.6)	0.12
Gestational week 28	110 (9)	110 (11)	0.6 (−2.3 to 3.4)	0.69
**Diastolic blood pressure, mm Hg (n=166)**				
Baseline	79 (7)	79 (6)	—	—
Prepregnancy week 8	77 (6)	77 (6)	0.0 (−2.1 to 2.0)	0.97
Gestational week 12	71 (8)	70 (6)	−0.9 (−3.1 to 1.3)	0.41
Gestational week 28	69 (8)	70 (8)	0.5 (−1.7 to 2.8)	0.65
**Resting heart rate, beats per minute (n=166)**				
Baseline	72 (11)	71 (11)	—	—
Prepregnancy week 8	69 (9)	67 (10)	−1.7 (−4.8 to 1.4)	0.29
Gestational week 12	71 (11)	69 (15)	−1.9 (−5.4 to 1.5)	0.26
Gestational week 28	78 (12)	77 (13)	−0.9 (−4.3 to 2.6)	0.63

Data are observed means and standard deviations at baseline, prepregnancy week 8, gestational week 12, and gestational week 28 for participants in each group. Results from linear mixed model analyses are presented as estimated mean difference (estimated effect) in intervention group compared with control group, with corresponding 95% confidence intervals and P values.

HbA1c=glycated haemoglobin; HDL=high density lipoprotein cholesterol; HOMA2-B=homoeostatic model assessment of β cell function; HOMA2-IR=homoeostatic model assessment of insulin resistance; LDL=low density lipoprotein cholesterol; OGTT=oral glucose tolerance test.

* 95% confidence intervals and P values are from bias corrected and accelerated confidence intervals based on bootstrap with 3000 samples owing to non-normally distributed residuals. Analysis for primary outcome included 102 (53 in control group, 49 in intervention group) of 109 participants because of missing outcome data at both time points (gestational week 12 and gestational week 28). For secondary outcome variables, number of participants with all outcome data missing for each variable ranged from 0 to 3 in control group and 0 to 1 in intervention group.

### Secondary outcomes

The estimated mean weight gain in the intervention group at gestational week 28 was 2.0 kg lower (95% confidence interval −3.3 to −0.8, P=0.002), and fat mass gain was 1.5 kg lower (−2.5 to −0.4, P=0.008) than the control group (table 2). There was little or no evidence of other between group differences during pregnancy.

### Adherence

**Fig 4:**
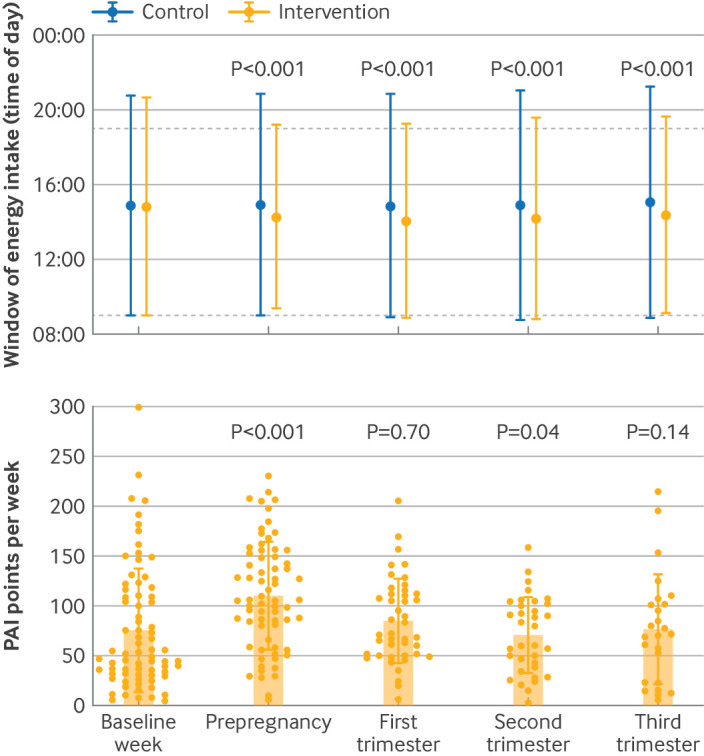
Adherence to intervention. Upper panel: time window of energy intake during baseline week, prepregnancy period, and first, second, and third trimesters of pregnancy according to group. Vertical lines represent daily duration of energy intake, with ends indicating means of time of first and last energy intake for intention-to-treat population. P values calculated for between group differences in window of energy intake using linear mixed model. Lower panel: physical activity intelligence (PAI) points earned for each rolling week during baseline week, prepregnancy period, and first, second, and third trimesters of pregnancy in intervention group. Bars show mean PAI points per week, error bars show standard deviation, and symbols show individual data. P values calculated for within group differences from baseline estimated by linear mixed model

At baseline, the average daily eating window for all participants was 11.9 hours (standard deviation 1.7). The eating window was shorter in the intervention group during the rest of the study period than in the control group (fig 4). In the prepregnancy period, the intervention group had an average eating window of 9.9 hours (standard deviation 1.2), which increased during pregnancy (fig 4 and supplementary table 2). In the prepregnancy period, 41/83 participants (49%) in the intervention group adhered to a ≤10 hour eating window. The proportion of pregnant participants who adhered to a ≤10 hour eating window was 23/55 (42%) in the first trimester, 17/55 (31%) in the second trimester, and 21/55 (38%) in the third trimester of pregnancy. The intervention group significantly increased their activity levels during the prepregnancy phase compared with baseline. The average weekly PAI points decreased throughout pregnancy and did not differ significantly from baseline (fig 4 and supplementary table 3). In the prepregnancy period, 36/83 participants (43%) obtained 100 weekly PAI points or more. The proportion of pregnant participants with at least 100 weekly PAI points was 16/55 (29%) in the first trimester, decreasing to 10/55 (18%) in the second trimester, and 8/55 (15%) in the third trimester of pregnancy.

### Per protocol analyses

In the per protocol analyses, we included only the participants in the intervention group who obtained ≥75 weekly PAI points and reported a ≤10 hour time window for energy intake on at least two of four days in the handbook in the prepregnancy period. Thirty one of 83 participants (37%) satisfied both criteria and were included in the analyses. Among the 55 pregnant participants in the intervention group, 24 (44%) were included in the per protocol analyses of outcomes specific to pregnancy (time to pregnancy and glycaemic outcomes during pregnancy). The results from the per protocol analyses were similar to those from the intention-to-treat analyses (supplementary tables 6-9), except for time to pregnancy which was significantly longer in the intervention group (48 days, 95% confidence interval 15 to 81, P=0.005; supplementary table 7).

### Dietary intake and physical activity

There was no statistically significant difference in self-reported total energy intake in the intervention group compared with the control group before pregnancy (−57.0 kcal/day, 95% confidence interval −183.9 to 69.9, P=0.38) or during the first trimester (41.2, −92.6 to 175.1, P=0.55), second trimester (39.7, –101.4 to 180.9, P=0.58), or third trimester (−110.3, −248.0 to 27.3, P=0.12). Additionally, there were no significant differences in the distribution of macronutrients in the intervention group compared with the control group before or during pregnancy (supplementary table 4). Self-reported physical activity level did not differ significantly between groups before or during pregnancy (supplementary table 5).

### Adverse events

No serious adverse events were reported during the study. Minor events included some participants feeling dizzy and nauseous during fasting blood sampling and the oral glucose tolerance test. Water intake and fresh air solved this problem for most participants, although some discontinued blood sampling. Some participants experienced local skin irritation from wearing a continuous glucose monitor, requiring premature removal. Others reported local skin irritation from the physical activity monitor armbands and smartwatches, which was resolved by repositioning the monitors or switching to metal wristwatch armbands.

## Discussion

### Main findings

The BEFORE THE BEGINNING study implemented a combination of time restricted eating and exercise training before conception and throughout pregnancy in people at increased risk of developing GDM. Contrary to our hypothesis, we found no significant between group differences in two hour plasma glucose level after a 75 g glucose load in gestational week 28, or in secondary outcome measures of glycaemic control at any time points before or during pregnancy. Although the intervention reduced body weight and fat mass gain at gestational week 28, there was no significant effect on GDM incidence. The participants were able to adhere to the ≤10 hour time restricted eating intervention before pregnancy, with a slight increase in the time window of energy intake during pregnancy. While participants in the intervention group earned more than 100 PAI points before pregnancy, their activity levels declined during pregnancy, and there were no significant between group differences in self-reported energy intake or physical activity throughout the study period.

### Strengths and limitations

The main strengths of the BEFORE THE BEGINNING trial are the randomised controlled design and longitudinal measurements throughout the prepregnancy and pregnancy periods. The study had a high pregnancy rate (110/166 participants, 66% in both groups), and a low dropout rate during pregnancy (8/109, 7% in both groups). However, the study has several limitations. Most participants were well educated, potentially resulting in healthy volunteer bias as they were likely knowledgeable about their health and wellbeing.^49^ Most of our participants were also white and so it is difficult to generalise our findings to other ethnicities. However, recruitment through social media is thought to give a better representation of the general population than other recruitment methods.^50^ Because we had a low number of participants (n=3) with previous GDM, adjustment for the stratification variable (GDM) was not included in the analyses. Despite good adherence to time restricted eating and exercise training in the prepregnancy period, we did not observe any improvements in cardiometabolic outcomes compared with the control group.

A previous trial of lifestyle intervention in pregnancy for people at high risk of GDM showed that individual changes in physical activity and dietary intake do not need to be large to have a beneficial effect on the incidence of GDM.^16^ The changes induced by the intervention in our trial could, however, be too small to affect our primary outcome measure. The declining adherence during pregnancy, along with a control group that was motivated to adopt a healthy lifestyle before pregnancy, are probably important reasons for this finding. Self-reported activity levels did not differ significantly between groups, suggesting that the level of physical activity in the control group was similar to the intervention group.

### Comparisons with other studies

Previous research indicated that the effectiveness of lifestyle interventions for GDM prevention is greater when they are started early in pregnancy, target high risk populations, limit gestational weight gain, and include moderate intensity exercise for 50-60 minutes twice weekly.^51^ Most lifestyle interventions for GDM prevention begin during pregnancy, missing the crucial window of opportunity before pregnancy to improve cardiometabolic health outcomes in those at risk.^15^ Systematic reviews and meta-analyses have highlighted the lack of randomised controlled trials focusing on lifestyle interventions before pregnancy in this population.^52^ ^53^ To date, only a handful of studies have investigated the impact of lifestyle interventions before pregnancy and continued throughout pregnancy on cardiometabolic outcomes.^25^ ^26^ ^54^ ^55^ ^56^ We found no significant effect of the intervention on glucose tolerance at gestational week 28, or on any other glycaemic indices. In contrast to our hypothesis, we estimated a higher mean two hour plasma glucose concentration in the intervention group at gestational week 28 according to the intention-to-treat and per protocol analysis (0.48 and 0.64 mmol/L, respectively) compared with the control group. However, the estimated mean differences were smaller than what was considered a clinically relevant difference (1.0 mmol/L). A slight, non-significant increase in two hour plasma glucose concentration (0.2 mmol/L) was also observed after a five week time restricted eating intervention during pregnancy.^32^

In contrast to our findings, Price and colleagues^55^ reported that a 12 week very low energy diet intervention before pregnancy significantly reduced two hour glucose during a 75 g oral glucose tolerance test by 0.8 mmol/L compared with a standard diet intervention group among women with body mass index between 30 and 55. In that study, the participants in the intervention group consumed approximately 800 kcal/day for 12 weeks, followed by a maintenance period of energy expenditure matched energy intake throughout the prepregnancy period, while also being advised to remain physically active (>10 000 steps/day). The 12 week prepregnancy intervention induced weight loss of 9.2 kg compared with the standard diet, but gestational weight gain remained unaffected.^55^ Based on the findings from Price and colleagues^55^ and our study, it seems likely that a prepregnancy intervention must induce substantial weight loss to impact glucose tolerance in pregnancy. Our trial, along with others,^26^ ^54^ ^55^ ^56^ did not find any significant effect of prepregnancy lifestyle interventions on GDM incidence at gestational week 28. However, GDM rates were lower in the intervention group in the Prepare trial in early pregnancy after a prepregnancy weight loss intervention.^25^ Despite being at higher risk of GDM, with 146/166 participants (86%) having a body mass index greater than 25 and 44/166 (27%) having a family history of diabetes, only 14/102 (14%) of our participants were diagnosed with GDM in gestational week 28. The GDM incidence in our trial was substantially lower than in the RADIEL study (60% in the control group and 54% in the intervention group)^54^ and in the study by Phelan and colleagues (40% in the control group and 25% in the intervention group).^56^ The low incidence of GDM observed in our study might be attributed to the high education level in 144/166 participants (87%) and 148/166 (89%) being white.^57^ All our participants included in the analyses were normoglycaemic at baseline, which could help explain the lack of effect because people with impaired glucose metabolism benefit the most from time restricted eating.^58^

There was a non-significant delay in the mean time to pregnancy in the intervention group (112 days) compared with the control group (84 days) in the intention-to-treat analysis. Six participants in the intervention group and three participants in the control group had a prolonged period in the trial because of spontaneous abortions. In our analysis, we estimated the time to pregnancy as the time from inclusion in the trial until the current pregnancy, and this was not always the first pregnancy after inclusion. Therefore, three participants in the intervention group were defined as pregnant more than one year after baseline (after 400, 397, and 374 days, respectively). We do not believe that the intervention affected fecundity. Evidence about the association between physical activity and fertility is inconsistent, but lifestyle interventions can increase natural conception in women with subfertility who are overweight or have obesity.^59^ Some studies indicate that large amounts of high intensity physical activity are negatively associated with fertility outcomes. However, an increased risk of infertility is typically only seen in those with the highest levels of intensity and frequency of physical activity. In our study, participants in the intervention group were asked to obtain at least 100 weekly PAI points, which is far less than the physical activity levels associated with reduced fertility outcomes in previous studies.

The intervention group had significantly lower body weight gain (2.0 kg) at gestational week 28 compared with the control group, but there were no significant differences between groups in the prepregnancy period or during the first trimester of pregnancy. In contrast, some of the previous prepregnancy lifestyle interventions induced weight loss before pregnancy, but did not affect weight gain during pregnancy.^26^ ^54^ ^55^ ^56^ In the Prepare study, the intervention group lost weight before pregnancy, followed by a greater weight gain in late pregnancy.^26^ The addition of exercise training to the time restricted eating intervention in our study might have prevented lean mass loss, which is common in time restricted eating interventions.^30^ Although not statistically significant, the estimated mean visceral fat area was numerically lower in the intervention group compared with the control group before and during pregnancy. The amount of visceral fat in early pregnancy has been shown to better predict GDM than body mass index before pregnancy.^60^ However, the observed changes in body composition might not have been large enough to improve the other cardiometabolic outcomes in our study.

There were no significant between group differences in total energy intake or macronutrient distribution before or during pregnancy, which could partly explain the neutral effect of the intervention on most outcome measures. In contrast, Haganes and colleagues^28^ reported that seven weeks of combined time restricted eating and HIIT induced a reduction in energy intake of approximately 200 kcal/day compared with the control group, along with improvements in several cardiometabolic outcomes, among reproductive aged women with overweight or obesity. Correspondingly, the combination of time restricted eating and high intensity functional training for 12 weeks resulted in a 175 kcal/day reduction in total energy intake among women with obesity.^61^ In BEFORE THE BEGINNING, we chose a modified time restricted eating regimen, allowing for unrestricted intake on two days per week. Even if such a regimen allows for more flexibility and potentially improved long term adherence than a stricter time restricted eating intervention, our intervention was not sufficiently potent to reduce energy intake. Participants undergoing dietary interventions often underreport their total energy intake,^62^ including when using the electronic application that we used.^63^ However, there is no reason to believe that such underreporting would be different between the intervention and control groups.

There is little research on the safety and acceptability of time restricted eating during pregnancy, with one previous randomised controlled trial on the effects of time restricted eating on glycaemic control.^32^ In a recent online survey study, only around half of the participants agreed that a time restricted eating pattern is safe during pregnancy and 23.7% were willing to try time restricted eating during pregnancy to improve their health.^64^ There was a gradual decrease in the adherence to time restricted eating in our study from before pregnancy and during pregnancy. In our previous study of time restricted eating in pregnancy,^32^ the participants could adhere to a 10 hour time restricted eating intervention on around five days per week for five weeks in the second or third trimester of pregnancy. However, in that study, the trial period was markedly shorter, and the participants were already pregnant at inclusion, and so likely less affected by nausea and other barriers to time restricted eating than in the BEFORE THE BEGINNING study. Collectively, the experimental evidence to date on time restricted eating during pregnancy indicates that there is no positive effect on maternal glycaemic control.

HIIT is now considered safe and feasible during pregnancy.^34^ ^35^ ^36^ However, the long term adherence to HIIT interventions during pregnancy remains to be explored. In our study, the participants in the intervention group substantially increased their physical activity levels before pregnancy with a decrease during pregnancy, suggesting suboptimal adherence to the exercise component of the intervention. Only 36/83 participants (43%) in the intervention group who became pregnant met the goal of 100 weekly PAI points before pregnancy, decreasing gradually to 8/55 (15%) during the third trimester. Although self-reported physical activity levels did not differ significantly between the groups, the reported physical activity levels were higher in the intervention group throughout the study. Adherence to exercise training is typically lower in unsupervised compared with supervised situations.^65^ The declining adherence to exercise during the study period was likely because of a combination of the unsupervised nature of the intervention, pregnancy related side effects (eg, nausea, pelvic pain), and decreasing motivation over a long study period. Overall, adherence to lifestyle interventions in pregnancy remains a significant challenge, especially in real life settings without close supervision.

### Conclusion and implications for clinicians and policy makers

The combination of time restricted eating and exercise training, started before and continued throughout pregnancy, had limited effects on glucose tolerance in late pregnancy among people with an increased risk of GDM. Despite the challenges with adherence to the intervention, the intervention group had lower weight and fat mass gain at gestational week 28. Emerging evidence highlights that the prepregnancy period is an ideal time to intervene in people at high risk of cardiometabolic diseases, and it is critical to find optimal strategies to improve adherence to lifestyle interventions in this population. Because lifestyle interventions are hard to implement, we need to know the minimum requirements for interventions to be effective and who will benefit the most from such interventions. GDM is a heterogeneous disease, and it could be relevant to distinguish between risk factors for GDM in the clinical care of people with increased risk of GDM.

### Unanswered questions and future research

We experienced challenges with adherence to the intervention, particularly the exercise training component in pregnancy. Future studies should assess whether more organised high intensity exercise training during pregnancy leads to better long term exercise adherence. Using more interactive digital health technology (eg, smartphone apps, automatically delivered daily reminders, weekly goals) should also be explored. The per protocol analysis indicated longer time to pregnancy in the intervention group, which should be further investigated. Not all participants in our study had high body mass index (some were included based on other risk factors), and therefore, there were variations in body composition and cardiometabolic markers. Future studies should also determine whether interventions based on single risk factors (eg, high body mass index, previous GDM), to avoid large within group variations, lead to better cardiometabolic outcomes.

**What is already known on this topic**

High body mass index before pregnancy and excessive gestational weight gain are associated with a greater risk of developing gestational diabetes mellitus and adverse pregnancy and neonatal outcomes
Systematic reviews and meta-analyses suggest that lifestyle interventions before pregnancy are necessary to improve maternal and fetal cardiometabolic outcomes
Existing guidelines on diet and physical activity during pregnancy are insufficient to achieve clinically significant cardiometabolic benefits

**What this study adds**

The BEFORE THE BEGINNING trial investigated the effect of combined time restricted eating and exercise training before and during pregnancy in people at high risk of gestational diabetes mellitus
The intervention did not result in significantly improved glycaemic outcomes
Some evidence was found of lower weight and fat mass gain at gestational week 28 in the intervention group than in the control group

## Data Availability

All individual deidentified participant data and statistical codes are available on Zenodo data repository. The code used to analyse the data in the paper can be found in the supplementary files. The data underlying the findings in this paper are openly and publicly available and can be found here: https://doi.org/10.5281/zenodo.15675472. For problems accessing the data, please contact the corresponding author.

## Web extra

Extra material supplied by authors

Web appendix: Supplementary materials

## Notes

Contributors: TM and MAJS had full access to all of the data in the study and take responsibility for the data integrity and the accuracy of the data analyses. TM, KÅS, TF, and SLF conceived and contributed to the study design and data analysis plans. GR, MAJS, and HMSS coordinated the study, performed measurements, monitored participants, and supervised the exercise training. MAJS and HMSS contributed equally to the work. All authors provided feedback and approved the final manuscript. TM is the guarantor for the paper and accepts full responsibility for the work and the conduct of the study, had access to the data, and controlled the decision to publish. The corresponding author attests that all listed authors meet authorship criteria and that no others meeting the criteria have been omitted.

Transparency: The lead author (the manuscript’s guarantor) affirms that the manuscript is an honest, accurate, and transparent account of the study being reported; that no important aspects of the study have been omitted; and that any discrepancies from the study as originally planned (and, if relevant, registered) have been explained.

Dissemination to participants and related patient and public communities: The authors plan to disseminate all the results to study participants and patient organisations as soon as the study is published. The results of this trial will be presented at relevant national and international conferences.

Provenance and peer review: Not commissioned; externally peer reviewed.
